# Transforming growth factor‐β‐mediated regulation of *atoh1*‐expressing neural progenitors is involved in the generation of cerebellar granule cells in larval and adult zebrafish

**DOI:** 10.1111/dgd.70002

**Published:** 2025-02-27

**Authors:** Jui Chun Wang, Takashi Shimizu, Masahiko Hibi

**Affiliations:** ^1^ Department of Biological Science, Graduate School of Science Nagoya University Nagoya Japan

**Keywords:** adult neurogenesis, *atoh1*, cerebellum, neural progenitor cells, zebrafish

## Abstract

Granule cells in the cerebellum are the most numerous neurons in the vertebrate brain. They are derived from neural progenitor cells that express the proneural gene *atoh1* (*atoh1a*, *b*, *c* in zebrafish) during early neurogenesis. In zebrafish, unlike in mammals, granule cells are continuously produced throughout life, from the larval stage to adulthood. Additionally, granule cells regenerate and replace damaged areas following injury in the adult cerebellum. However, the mechanisms underlying granule cell generation and their role in adult cerebellar regeneration remain largely unclear. In this study, using lineage tracing with the inducible DNA recombinase CreERT2, we found that granule cells differentiated from *atoh1c*‐expressing neural progenitor cells and migrated to their appropriate locations in the adult stage, similar to the processes observed during early embryogenesis. Granule cells that differentiated from *atoh1c*‐expressing neural progenitor cells in adulthood also contributed to cerebellar regeneration. Furthermore, inhibition of transforming growth factor‐β (TGF‐β) signaling, either via chemical inhibitors or CRISPR/Cas9, suppressed *atoh1a/c* expression and reduced granule cell numbers in larvae. Chemical inhibition of TGF‐β signaling also suppressed neural progenitor cell proliferation, *atoh1c* expression, and granule cell neurogenesis in the adult cerebellum. These findings demonstrate that TGF‐β signaling is essential for granule cell production from progenitor cells throughout the lifespan of zebrafish.

## Graphical abstract

Proliferating neural progenitor cells that generate granule cells are located in the molecular layer (ML) of the cerebellum. Among these, some atoh1c‐expressing cells begin to differentiate and migrate to the granular layer (GL), where they localize as mature granule cells. A similar process occurs in the valvula cerebelli (Va), the anterior part of the cerebellum. In the torus longitudinalis (TL) of the mesencephalon, granule cells are derived from atoh1c‐expressing cells in larvae, but their production is limited in adults.

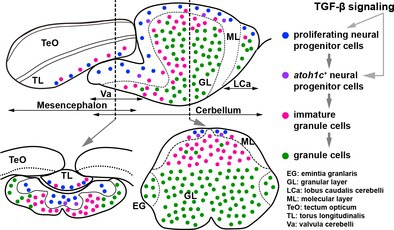

## 1 INTRODUCTION

**FIGURE 1:**
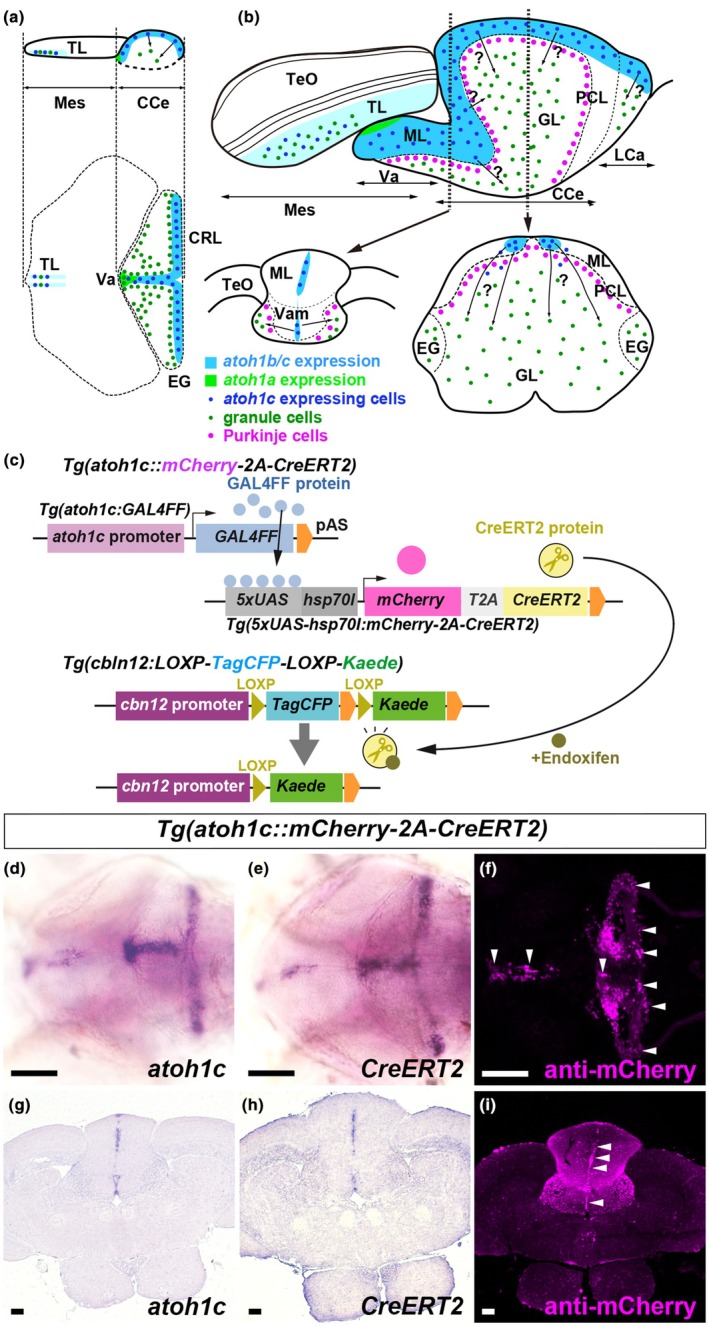
Tracing of *atoh1c*‐expressing cell lineage. (a, b) Schematic of the cerebellum and dorsal mesencephalon in larval (5 dpf, a) and adult (b) zebrafish. Sagittal section (upper) and dorsal view (lower) images in (a), while sagittal (upper) and two cross‐section (lower) views are shown in (b)—one is at the boundary between the Va and CCe and the other is at the middle of the CCe). CCe, corpus cerebelli; CRL, cerebellar rhombic lip; EG, eminentia granularis; GL, granular layer; LCa, lobus caudalis cerebelli; Mes, mesencephalon; ML, molecular layer; PCL, Purkinje cell layer; TeO, tectum opticum; TL, torus longitudinalis; Va, valvula cerebelli; Vam, medial division of valvula cerebelli. (c) Lineage tracing system. *Tg*(*atoh1c::mCherry‐2A‐CreERT2*) fish harbor a transgene that expresses *GAL4FF* under the control of the *atoh1c* promoter (*Tg*(*atoh1c:GAL4FF*)), and a transgene that expresses mCherry and CreERT2 under the control of GAL4 binding sites (UAS, upstream activating sequence) and the zebrafish *hsp70l* promoter (*Tg*(*5xUAS‐hsp70l:mCherry‐2A‐TagCFP*)). *Tg*(*cbln12:LOXP‐TagCFP‐LOXP‐Kaede*) fish express TagCFP in the cerebellar GCs under the control of the promoter of the *cbln12* promoter, which is specific to GCs, and express Kaede upon CreERT2/*LOXP*‐mediated recombination. When fish from the cross of *Tg*(*atoh1c::mCherry‐2A‐CreERT2*) and *Tg*(*cbln12:LOXP‐TagCFP‐LOXP‐Kaede*) are treated with endoxifen, the GCs derived from *atoh1c*‐expressing neural progenitors are marked by Kaede expression. (d–f) Expression of *atoh1c*, *CreERT2* and mCherry in 5‐dpf *Tg*(*atoh1c::mCherry‐2A‐CreERT*) larvae. Dorsal views with anterior to the left. (g–i) Expression of *atoh1c*, *CreERT2*, and mCherry in cross‐sections of the rostral region of the cerebellum (corresponding to the lower‐left panel in b) in adult *Tg*(*atoh1c::mCherry‐2A‐CreERT*) fish. Dorsal is oriented upwards. In situ hybridization (d, e, g, h). Immunostaining (f, i). mCherry expression in the *atoh1c*/CreERT2‐expressing regions is marked by arrowheads. Scale bars: 100 μm in d (applies to d–f), 100 μm in g (applies to g–i).

The cerebellum, located in the dorsal anterior region of the hindbrain, plays a role in various functions including motor learning, motor coordination, cognitive functions, and emotional responses (Koziol et al., 2014; Leto et al., 2016). The functions of the cerebellum rely on its structure and its connected neural circuits, which are conserved among vertebrates (Hashimoto & Hibi, 2012; Hibi et al., 2017; Hibi & Shimizu, 2012). In both teleosts and mammals, cerebellar neurons can be classified into two groups based on their primary neurotransmitter: glutamatergic or GABAergic (Altman & Bayer, 1997; Bae et al., 2009; Butler & Hodos, 2005). Granule cells (GCs) are a major subset of glutamatergic neurons, accounting for more than half of the total neuronal population in the brain, while Purkinje cells (PCs) are a key subset of GABAergic neurons in the cerebellum (Galliano et al., 2013; Lange, 1975). Cerebellar neurons, including GCs and PCs, form a three‐layered structure in the zebrafish cerebellum (Figure 1a,b) (Bae et al., 2009). From the surface inward, these layers are the molecular layer (ML), Purkinje cell layer (PCL), and the granular layer (GL). The somata of differentiated GCs and PCs are located in the GL and PCL, respectively, while the axons of GCs and the dendrites of PCs form neural circuits (e.g. synapses) in the ML. Additionally, the cerebellum consists of four lobes: the most anterior is the valvula cerebelli (Va), the central part is the corpus cerebelli (CCe), and the posterior and lateral regions are called the lobus caudalis cerebelli (LCa) and eminentia granularis (EG). The Va and CCe exhibit the typical three‐layered structure, whereas the LCa and EG lack the PCL and ML on their surface. In teleosts, in addition to the cerebellum, there are neural structures known as cerebellum‐like structures, which have a similar neural circuitry to the cerebellum. The torus longitudinalis (TL), one of these cerebellum‐like structures located in the dorsal midbrain, contains numerous GCs and forms neural circuits with PC‐like cells (type I neurons) in the tectum opticum (TeO) (Bae et al., 2009; Nimura et al., 2019; Wullimann, 1994).

Genetic studies in mice have shown that GCs originate from neural progenitors located in the cerebellar rhombic lip (CRL, also referred to as the upper rhombic lip). These progenitors express the proneural gene *Atoh1* (also known as *Math1* in mice) (Alder et al., 1996; Ben‐Arie et al., 1997; Machold & Fishell, 2005; Wang et al., 2005). These progenitor cells proliferate and expand on the dorsal surface of the cerebellum, forming the external granular layer (EGL) (Butts et al., 2014). During the development, some of these neural progenitors cease cell proliferation, differentiate into GCs, and migrate inward (ventrally) to form the GL (also referred to as the internal granular layer) (Machold & Fishell, 2005; Wang et al., 2005). During this process, the differentiated cells express the basic helix–loop–helix gene *Neurod1*, which has been shown to be essential for GC differentiation (Miyata et al., 1999).

Zebrafish have three *atoh1* paralogs: *atoh1a*, *atoh1b*, and *atoh1c*. These genes are expressed in overlapping but distinct regions of the CRL in early‐stage larvae starting at 2 days post fertilization (dpf) (Adolf et al., 2004; Chaplin et al., 2010; Kani et al., 2010) (Figure 1a). In addition to the CRL in the cerebellum, *atoh1c* is also expressed in the TL starting at 3 dpf (Kani et al., 2010). At least some of the *atoh1a/b/c*‐expressing cells proliferate and give rise to Neurod1‐expressing GCs, which migrate to the GL in the Va and CCe, or remain in the LCa and EG (Kani et al., 2010; Volkmann et al., 2008). Although these *atoh1*‐expressing cells do not form a typical EGL as seen in mammals, these findings suggest that they function as neural progenitors for GCs in larvae. Among *atoh1a/b/c*, *atoh1a* and *atoh1c* function redundantly, with *atoh1c* playing a predominant role in the differentiation of GCs, as *atoh1c* mutant larvae show a significant reduction in the GC marker expression (Kidwell et al., 2018). In zebrafish, GCs are continuously generated from neural progenitors proliferating in the ML throughout life, and they migrate into the GL (Kani et al., 2010; Kaslin et al., 2009; Kaslin et al., 2013; Zupanc et al., 2005). *atoh1a/b/c*‐expressing cells are present in the medial region of the ML of the Va and CCe, as well as on the dorsomedial surface of the LCa in the adult cerebellum, and have been shown to proliferate (Kani et al., 2010) (Figure 1b). Additionally, studies using transgenic (Tg) *nestin:egfp* zebrafish demonstrated that *nestin*‐expressing cells and their progeny give rise to GCs in the adult cerebellum (Kaslin et al., 2009; Kaslin et al., 2013). Therefore, it remains unclear whether *atoh1*‐expressing cells in the adult cerebellum also function as neural progenitors for GCs during adulthood.

In amniotes (mammals and birds), the proliferation of GC progenitors in the EGL is positively regulated by Sonic hedgehog (Shh), which is secreted by PCs (Dahmane & Ruiz i Altaba, 1999; Lewis et al., 2004; Wallace, 1999; Wechsler‐Reya & Scott, 1999). Zebrafish have two *shh* orthologs, *shha* and *shhb* (previously known as *tiggy winkle hedgehog*, *twhh*) (https://zfin.org). Although *shha* has been reported to be expressed in the eurydendroid cells, which are the output neurons of the cerebellum (Biechl et al., 2016), it is not expressed in PCs, and *ptch1* (*patched1*), which is a target of Shh signaling, is not expressed in the larval cerebellum (Chaplin et al., 2010). RNA‐seqencing analysis further revealed that *shha* or *shhb* expression is negligibly detected in PCs in the larval cerebellum (Takeuchi et al., 2017). Additionally, *shha* promoter activity is not detected in the adult cerebellum (Wullimann & Umeasalugo, 2020). Yet, the role of Shh signaling in the formation and maintenance of *atoh1*‐expressing neural progenitors has not yet been formally investigated. In addition to Shh, growth differentiation factors 7 and 6a (*gdf7* and *gdf6a*), which encode transforming growth factor‐β (TGF‐β) ‐family cytokines that activate bone morphogenetic protein (BMP) signaling, are expressed at near *atoh1a/b*‐ and *atoh1c*‐expressing neural progenitors, respectively (Chaplin et al., 2010), suggesting a role for BMP signaling in the formation and/or maintenance of *atoh1*‐expressing GC progenitors. In chick embryos, *gdf7* is also expressed in the CRL during cerebellar development (Broom et al., 2012; Green et al., 2014). However, inhibition of BMP signaling by overexpressing Smad6 in chick embryos does not affect the formation of mature GCs but rather impairs the migration of GC progenitors (Rook et al., 2024), suggesting that BMP signaling may not be involved in the formation and maintenance of GC progenitors. Furthermore, inhibition of fibroblast growth factor (Fgf) signaling by expressing a dominant‐negative Fgf receptor 1 inhibits the proliferation of neural progenitors in the medial ML of the adult cerebellum (Kaslin et al., 2009), implying a role for Fgf signaling in the maintenance of GC progenitors in adulthood. Fgf signaling has also been reported to be involved in the formation and/or maintenance of the *Atoh1*‐expressing CRL domain in chick and mouse embryos (Green et al., 2014). Although many candidate signaling molecules involved in the formation and/or maintenance of GC progenitors have been identified, their precise roles, as well as the functions of other potential signaling molecules, remain unclear.

In mammals, neuronal regeneration in the vertebrate brain has been observed, but it generally occurs with limited capacity and is restricted to specific brain regions (Iismaa et al., 2018). Conversely, in some amphibians and teleosts, neuronal regeneration in various parts of the brain has been observed not only during early developmental stages or in juveniles, but also in adulthood (Hentig et al., 2021; Kizil et al., 2012; Lust & Tanaka, 2019; Tanaka & Reddien, 2011; Zambusi & Ninkovic, 2020). In the zebrafish cerebellum, when the cerebellar region is ablated during early development, GCs regenerate within a few days (Köster & Fraser, 2006). It has also been shown that in adult zebrafish, following cerebellar injury, neural progenitors in the medial molecular layer (ML) proliferate, regenerating GCs and restoring their functional capacity (Hentig et al., 2021; Kaslin et al., 2017). However, it remains unclear whether *atoh1*‐expressing cells function as GC neural progenitors during GC regeneration and what signaling pathways are involved in this process.

In this study, we analyzed the contribution of *atoh1*‐expressing neural progenitor cells to adult neurogenesis and the regeneration of GCs. Additionally, we investigated the intercellular signaling involved in the formation and maintenance of *atoh1*‐expressing neural progenitor cells.

## 2 MATERIALS AND METHODS

### 2.1 Ethics declarations

The animal work in this study was approved by the Nagoya University Animal Experiment Committee and was conducted in accordance with the Regulations on Animal Experiments at Nagoya University.

### 2.2 Zebrafish strains

Wild‐type zebrafish with the Oregon AB genetic background were used. For immunohistochemistry and whole‐mount in situ hybridization, larvae were treated with 0.003% 1‐phenyl‐2‐thiourea (PTU) (Nacalai‐Tesque, 27429‐22) to inhibit the formation of pigmentation. Transgenic zebrafish *TgBAC*(*atoh1c:GAL4FF*)*fh430Tg*, *Tg*(*UAS‐E1B:Kaede*)*s1999tTg* (Kidwell et al., 2018), *Tg*(*5xUAS‐hsp70l:mCherry‐2A‐CreERT2*)*nub99Tg*, *Tg*(*cbln12:LOXP‐TagCFP‐LOXP‐Kaede*)*nub96Tg* (Itoh et al., 2024), and *TgBAC*(*atoh1a:EGFP*)*nns7* (Satou et al., 2013) were previously described. Zebrafish were maintained at 28°C under a 14‐h light and 10‐h dark cycle. Embryos and larvae were maintained in embryonic medium (Westerfield, 2000). In this study, fish aged 3–6 months post‐fertilization were used as adults.

### 2.3 Lineage tracing

To treat larvae, a 4‐μM endoxifen solution was prepared by adding 0.96 μL of 25 mM endoxifen (Sigma‐Aldrich, SML2368) dissolved in dimethyl sulfoxide (DMSO) to 6 mL of E3 medium (5 mM NaCl, 0.17 mM KCl, 0.4 mM CaCl_2_, and 0.16 mM MgSO_4_) with 0.004% PTU. Larvae at 2 dpf were treated for 16 h to induce CreERT2‐mediated recombination, then maintained in E3/PTU medium until 5 dpf. For adult fish, a 4‐μM endoxifen solution was prepared similarly in 48 mL of system water. Fish were treated for 16 h, rinsed twice, and returned to the rearing tanks. DMSO alone was used as a control.

### 2.4 Immunostaining

For immunostaining, anti‐Neurod1 (1:500, mouse ascites) (Kani et al., 2010), anti‐GFP (recognizes TagCFP, 1:1000, rat, Nacalai‐Tesque, 04404–84), anti‐DsRed (recognizes mCherry, 1:1000, rabbit, Clontech Laboratories, 632496), anti‐bromodeoxyuridine (BrdU) (1:500, rat, Abcam, ab6326), and anti‐Kaede (1:500, rabbit, MBL, M‐106‐3 M) antibodies were used. CF488A goat anti‐mouse IgG (H + L, Biotium, 20,018), CF488A goat anti‐mouse IgG (H + L, Biotium, 20012), CF488A goat anti‐rat IgG (H + L, Biotium, 20023), CF488A goat anti‐rabbit IgG (H + L, Biotium, 20102), CF568 goat anti‐rabbit IgG (H + L, Biotium, 20018), and Alexa Fluor 405 goat anti‐rat IgG (H&L, Abcam, ab175673) were used as the secondary antibodies. Larvae and cryosections were immunostained as previously described (Bae et al., 2009), with 14‐μm sections prepared using a cryostat HM525NX (PHC). Fluorescence images were captured with an LSM700 confocal laser‐scanning microscope or an Axio Imager microscope/AxioCam CCD camera (Zeiss). Projection images were constructed from Z‐stack sections using the three‐dimensional‐projection program associated with the microscope (ZEN, Zeiss). Figures were assembled using Adobe Photoshop, and Adobe Illustrator, with brightness and contrast adjustments applied equally to all digital images within each figure.

### 2.5 BrdU incorporation

BrdU incorporation was performed with slight modifications to the protocol previously described (Kani et al., 2010). Briefly, adult fish were incubated in a 1% BrdU (5′‐bromo‐deoxyuridine, Fujifilm Wako) solution for 1 hour. Brains were then extracted and fixed in 4% paraformaldehyde in phosphate‐buffered saline (PBS) for 16 h. Brain cryosections were treated with 2 N HCl for 30 min at room temperature, washed three times with PBS, 0.1% Triton X‐100, neutralized three times with 0.1 M Na_2_B_4_O_4_, and washed three times with PBS 0.1% Triton X‐100. Immunostaining was conducted as described above.

### 2.6 In situ hybridization

Whole‐mount in situ hybridization was performed as previously described (Bae et al., 2009). Detection of *ato1a*, *atoh1b*, and *atoh1c* was previously described (Kani et al., 2010). To generate the *CreERT2* riboprobe, *CreERT2* cDNA from pCre‐ERT2 (a gift from Pierre Chambon) (Feil et al., 1997; Indra et al., 1999) was subcloned into the *Eco*RI site of pCS2^+^. A digoxigenin (DIG)‐labeled riboprobe was synthesized using T7 RNA polymerase following digestion with *Hin*dIII. Larvae were hybridized with DIG‐labeled riboprobes overnight at 55°C and incubated overnight with 1/5000 alkaline phosphatase‐conjugated anti‐DIG Fab fragment (Roche, 11093274910) at 4°C. BM Purple AP substrate (Roche, 11442074001) was used as the alkaline phosphatase substrate. Images were acquired using an Axio Imager microscope equipped with an AxioCam CCD camera. For in situ hybridization of adult brain sections, the brains were dissected from adult zebrafish and fixed overnight at 4°C in 4% paraformaldehyde in PBS. The specimens were immersed in 30% sucrose solution overnight at 4°C, frozen in OCT compound (Sakura Finetechnical), and sectioned at 14 μm on a cryostat. The frozen sections were washed three times with Tris‐buffered saline (TBS) in diethylpyrocarbonate (DEPC)‐treated water for 10 min, treated with 0.2 M HCl for 10 min, and washed three times with TBS in DEPC‐treated water. They were subsequently treated with 0.1 M Tris–HCl (pH 8.0) containing acetic anhydride for 20 min and washed three times with TBS in DEPC‐treated water. They were incubated in hybridization buffer (50% formamide, 5× standard saline citrate [SSC], 50 μg/mL heparin, 0.3% Tween‐20, 5 mg/mL yeast torula RNA) for over 1 h and hybridized overnight with a DIG‐UTP‐labeled riboprobe at 55°C for *CreERT2* and 65°C for *atoh1c* detection. The specimens were quickly washed with 50% formamide 2× SSC at 65°C first and washed with 50% formamide 2× SSC at 65°C, 50% formamide 1× SSC, and 20% formamide 0.5× SSC for 30 min each. They were then washed with PBS, 0.3% Triton X‐100 (PBST) three times for 10 min, blocked with blocking buffer (1× Roche blocking reagent, 5% heat‐inactivated fetal bovine serum) for 30 min, and incubated with 1/5000 diluted alkaline phosphatase‐conjugated anti‐DIG in blocking buffer overnight at 4°C. After three washes with PBST, the sections were incubated in 100 mM NaCl, 100 mM Tris–HCl, pH 9.5, 50 mM MgCl_2_, 0.3% Triton X‐100 for 15 min. The detection of alkaline phosphatase signals and imaging were performed as described above.

### 2.7 Injury and regeneration

Endoxifen treatment was administered as described in Section 2.3 before wounding. Cerebellar injury was performed manually under anesthesia with tricaine (MS‐222, Nacalai‐Tesque 14805‐24) using a steel drill (Minitor, BS1201). The drill was inserted from the posterior edge of the supraoccipital bone and stopped at the anterior edge of the parietal bone, creating a column lesion approximately 300 μm in diameter through the cerebellum. Post‐surgery, the fish was moved to a separate tank for monitoring. Fish in the 5‐day post‐lesion group were killed on day 5, while those in the 3‐month post‐lesion group were transferred back to the main system on day 7 and maintained separately for 3 months.

### 2.8 Chemical inhibition

For chemical inhibition in larvae, 10 μM inhibitor solutions were prepared by adding 10 mM stocks of LDN193189 (MedChemExpress, HY‐12071, in DMSO), cyclopamine (Wako, 038–19,311, in ethanol), or vactosertib (MedChemExpress, HY‐11928, in DMSO) to E3/PTU medium. Larvae at 2 dpf were treated until 5 dpf. For adult fish, 10 μM vactosertib was prepared by adding 48 μL of vactosertib in DMSO to 48 mL of system water. Adult fish (3–6 months) were treated for 24 h, followed by 16 h with 7.68 μL of 25 mM endoxifen. After two washes, fish resumed vactosertib treatment for 4 days and 8 h. DMSO alone served as a control.

### 2.9 Crispants

To make crispants for *tgfbr1a* (*alk5a*) and *tgfbr1b* (*alk5b*), chemically synthesized Alt‐R crRNAs and tracrRNAs, and Cas9 protein (Integrated DNA Technologies) were used. The following target sequence was selected: 5′‐AATCGGTCGTGCAACACAAC(TGG)‐3′ for *tgfbr1a* and 5′‐CATGACGGTCTGGTAGATCT(CGG)‐3′ for *tgfbr1b* (PAM sequences are indicated in parentheses). crRNA for zebrafish *mitfb*, targeting 5′‐GAACAAGGGCACGATTCTGA(AGG)‐3′ was used for control crispants (Miyadai et al., 2023). To prepare the crRNA:tracrRNA Duplex and gRNA, Cas9 RNP complexes were established as previously reported (Hoshijima et al., 2019). A solution was prepared containing 5 μM crRNA, 5 μM tracrRNA and 5 μM Cas9 proteins. One nanoliter of the respective solution was injected into one‐cell‐stage embryos. To detect mutations, the target genomic regions were amplified by polymerase chain reaction with the following primers: 5′‐TCCAAGGACTTGTCCAACCG‐3′ and 5′‐CTCTGCTTGTCTAGCCCTGAT‐3′ for *tgfbr1a*, and 5′‐CTTCAAACGGCTGGCAAGTG‐3′ and 5′‐TATTGCGGGACAAACCTGCT‐3′ for *tgfbr1b*. The polymerase chain reaction products were separated on 12% Tris‐borate‐EDTA acrylamide gels.

### 2.10 Quantification of image data

To quantify marker‐positive cells in larvae, 1‐μm‐thick serial digital z‐stack sections were acquired using an LSM700 confocal laser‐scanning microscope through the entire cerebellum of zebrafish larvae. Marker‐positive cells were manually counted using each section image generated by ZEN software (Zeiss). The total count of positive cells in each larva was then used for statistical analysis.

### 2.11 Statistics

Data were analyzed using GraphPad Prism (version 5.1).

## 3 RESULTS

### 3.1 Lineage tracing of *atoh1c*‐expressing cell lineage

We performed lineage tracing using the CreERT2‐loxP system to analyze the process by which *atoh1c*‐expressing (*atoh1c* ^+^) neural progenitor cells differentiate into GCs (Figure 1c). We used the GAL4‐UAS system and viral 2A peptide to express mCherry and CreERT2 in *atoh1c* ^+^ cells, employing Tg lines *TgBAC*(*atoh1c:GAL4FF*) (GAL4FF is a modified version of GAL4‐VP16) (Kidwell et al., 2018) and *Tg*(*UAS‐hsp70l:mCherry‐2A‐CreERT2*) (Itoh et al., 2024) (referred to as *Tg*(*atoh1c::mCherry‐2A‐CreERT2*)). We compared the expression of *atoh1c*, *CreERT2* mRNAs, and mCherry protein in early‐stage (5 dpf) larvae. *CreERT2* expression recapitulated that of *atoh1c* in the TL and CRL (Figure 1d, e), whereas mCherry was predominantly detected in differentiated GCs in the CCe, as well as in the TL and CRL (Figure 1f). The data are consistent with the previous findings observed in *Tg*(*atoh1c::Kaede*) (Kidwell et al., 2018). Similarly, *CreERT2* mRNA was detected in the rostro‐medial region of the ML in adult zebrafish, where *atoh1c* is expressed (Figure 1g, h), whereas mCherry was more frequently detected in GCs compared with *atoh1c*‐expressing regions (Figure 1i). These findings indicate that, in this lineage tracing, *CreERT2* is expressed in *atoh1c* ^+^ cells in the CRL, leading to the translation of *CreERT2*‐2A‐*mCherry* mRNA and the subsequent detection of mCherry protein. As mCherry protein is more stable than *CreERT2*‐2A‐*mCherry* mRNA, mCherry likely marks CreERT2‐expressing cells transitioning from neural progenitor cells to differentiating GCs. We crossed *Tg*(*atoh1c::mCherry‐2A‐CreERT2*) with the reporter line *Tg*(*cbln12:LOXP‐TagCFP‐LOXP‐Kaede*), which expresses TagCFP in GCs in a *cbln12* promoter‐dependent manner (Dohaku et al., 2019; Itoh et al., 2024). In this experiment, activation of CreERT2 with endoxifen induced recombination of the reporter gene, resulting in the conversion of TagCFP to Kaede expression in GCs (Figure 1c).

### 3.2 *atoh1c*‐expressing progenitor cells give rise to GCs in larval and adult stages

**FIGURE 2:**
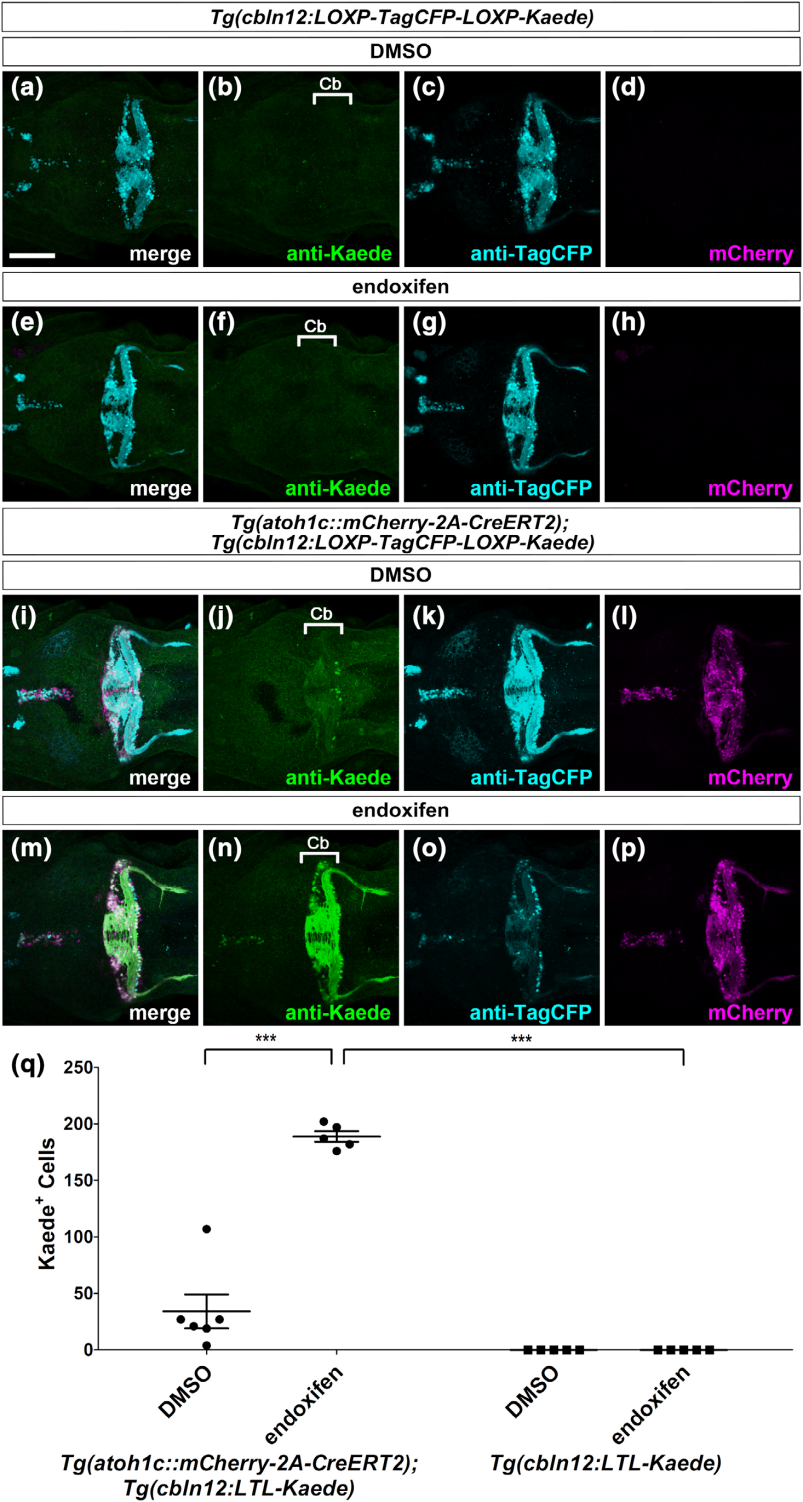
Granule cells are generated from *atoh1c*‐expressing neural progenitors in larvae. Expression of Kaede, TagCFP, and mCherry in 5‐dpf *Tg*(*cbln12:LOXP‐TagCFP‐LOXP‐Kaede*) (a–h) and *Tg*(*atoh1c::mCherry‐2A‐CreERT2*);*Tg*(*cbln12:LOXP‐TagCFP‐LOXP‐Kaede*) (i‐p) larvae treated with DMSO (a–d, i–l) or endoxifen (e–h, m–p) from 2 dpf. Kaede (b, f, j, n) and TagCFP (c, g, k, o) are visualized by immunostaining signals, while mCherry (d, h, l, p) shows its own fluorescence. Merged images are also shown (a, e, i, m). (q) Number of Kaede^+^ cells in each larva (*n =* 5 each condition). Kaede^+^ cells were counted in the cerebellar region, labeled as Cb in (b, f, j, n). One‐way analysis of variance with Tukey's post hoc test. *p < .05, ***p < .001. Scale bar: 100 μm in a (applies to a–p).

We first treated embryos from the crosses with either endoxifen or DMSO (control) at 2 dpf and examined marker expression at 5 dpf. In Tg larvae containing only the reporter gene, Kaede^+^ cells were barely observed in both control and endoxifen‐treated larvae (Figure 2b, f). In contrast, in Tg larvae containing both CreERT2 and reporter genes, only a few Kaede^+^ cells were observed in the cerebellum without endoxifen treatment (Figure 2j), indicating minimal leakiness of the reporter system. Following endoxifen treatment, Kaede^+^ cells appeared in the anterior TL, and a substantial number of Kaede^+^ cells were observed in the GL of the cerebellum (Figure 2n, q). These Kaede^+^ cells in the GL extended parallel fibers within and outside the cerebellum (Figure 2n). TagCFP signals were reduced in endoxifen‐treated embryos (Figure 2k, o), confirming CreERT2‐mediated recombination. These findings indicate that at least a portion of GCs are derived from *atoh1c* ^+^ cells in the TL and cerebellum of early‐stage zebrafish larvae.

**FIGURE 3:**
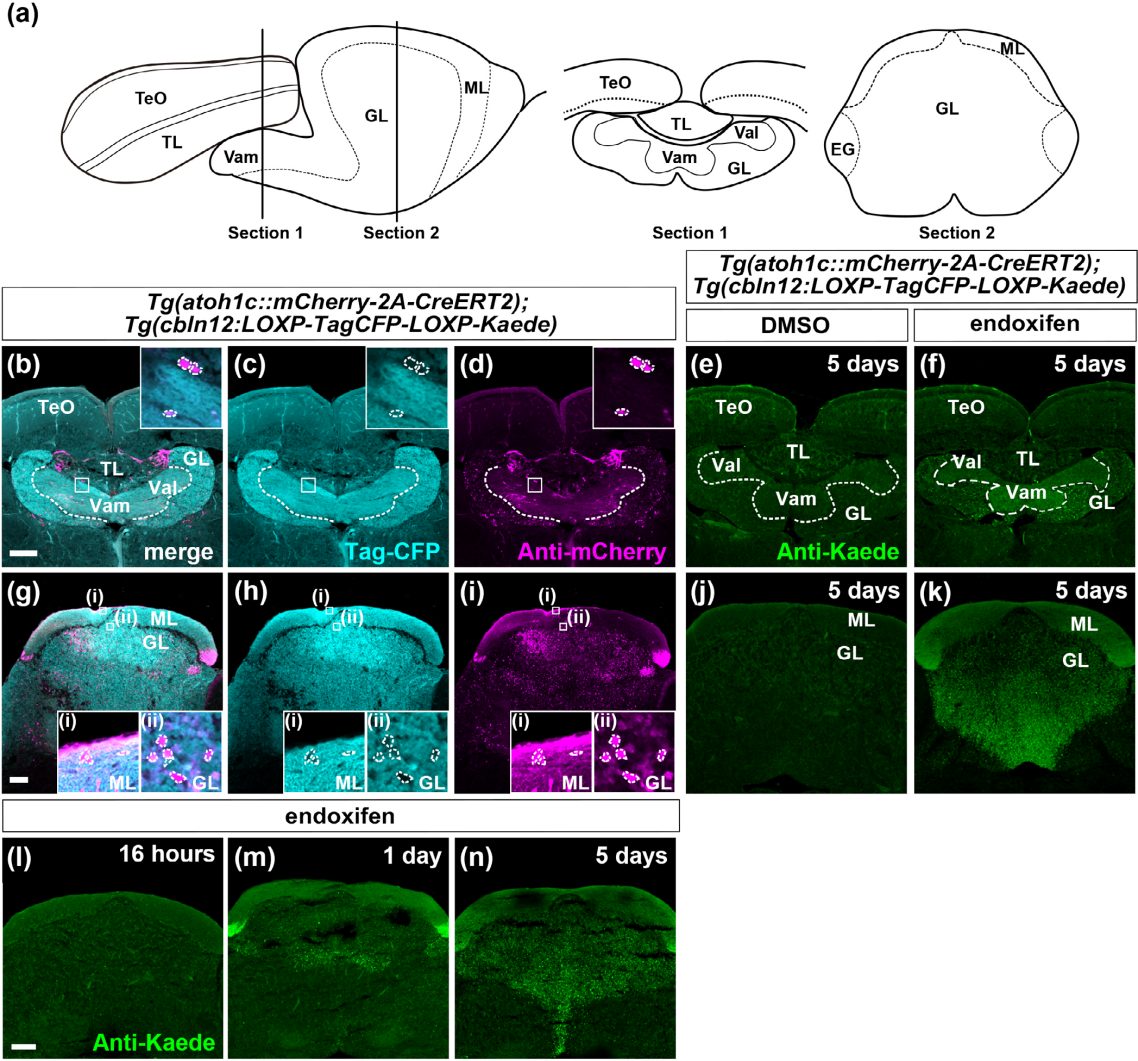
Granule cells are generated from *atoh1c*‐expressing neural progenitors in adult zebrafish. (a) Images of analyzed sections. Medial sagittal sections (left panel) and cross‐sections at two distinct anterior–posterior levels (section 1: TL/Va level; section 2: CCe level) were analyzed. Similar section levels were used for Figures 3, 4, 5, and 7. (b–d, g–i) Expression of TagCFP and mCherry in cross‐section 1 (b–d) and cross‐section 2 (g–i) of adult *Tg*(*atoh1c::mCherry‐2A‐CreERT2*);*Tg*(*cbln12:LOXP‐TagCFP‐LOXP‐Kaede*) fish (*n* = 4). Immunostaining. Higher magnification views of the boxed areas are shown in the insets in b–d and g–i. Cells outlined with dotted lines indicate mCherry^+^, TagCFP^−^ cells. (e, f, j, k, l–n) Tracing of *atoh1c*‐expressing cell lineage. Fish were treated with DMSO or endoxifen, and Kaede expression was analyzed at 5 days (e, f, j, k, n) (*n* = 5), 16 h (l) (*n* = 2), and 1 day (m) (*n* = 2) after treatment initiation. Immunostaining. Dotted lines indicate the boundary between Vam/Val and GL in b–f. Val, lateral division of valvula cerebelli. Other abbreviations are explained in the legend of Figure 1. Scale bars: 100 μm in b (applies to b–f), 100 μm in g (applies to g–k), 100 μm in l (applies to l–n).

We further conducted lineage tracing in adult zebrafish, analyzing marker expression at two anterior–posterior cross‐sectional levels: the TL/Va level (section 1) and the CCe level (section 2, Figure 3a). TagCFP signals were detected weakly in GCs in the TL and strongly in the somata and axons of GCs within both the ML (Vam/Val) and GL of the Va and CCe (Figure 3c, h), indicating that TagCFP is expressed in differentiated GCs. mCherry was detected in a small number of cells in the TL and in a relatively large number of Vam and Val (corresponding to the ML in the CCe) and GL of the Va (Figure 3d). In the CCe, mCherry was detected in a small number of cells in the ML and a relatively large number of cells in the dorsal GL, close to the ML (Figure 3i). These mCherry^+^ cells, which likely expressed CreERT2, were negative for TagCFP signals (see insets of Figure 3g–i), suggesting that they are differentiating neural progenitors or immature GCs that have not yet fully differentiated. To assess Kaede expression, we treated Tg adult fish with endoxifen or DMSO (control) and analyzed the expression at 5 days post‐treatment. Kaede was scarcely detected in control fish, but Kaede signals were observed in the somata of GCs within the Va and CCe regions of endoxifen‐treated fish, with minimal detection in the TL (Figure 3e–k). Kaede signal was also present in the ML, marking GC axons (Figure 3f, k). A time–course analysis of the lineage tracing in the CCe showed that while Kaede signals did not appear at 16 h, Kaede^+^ cells were observed at 1 day in the dorsal GL and later expanded throughout the GL (Figure 3l–n). These findings indicate that at least some GCs in the adult cerebellum are derived from *atoh1c* ^+^ neural progenitor cells. These GCs subsequently migrate to appropriate positions throughout the GL within 5 days.

**FIGURE 4:**
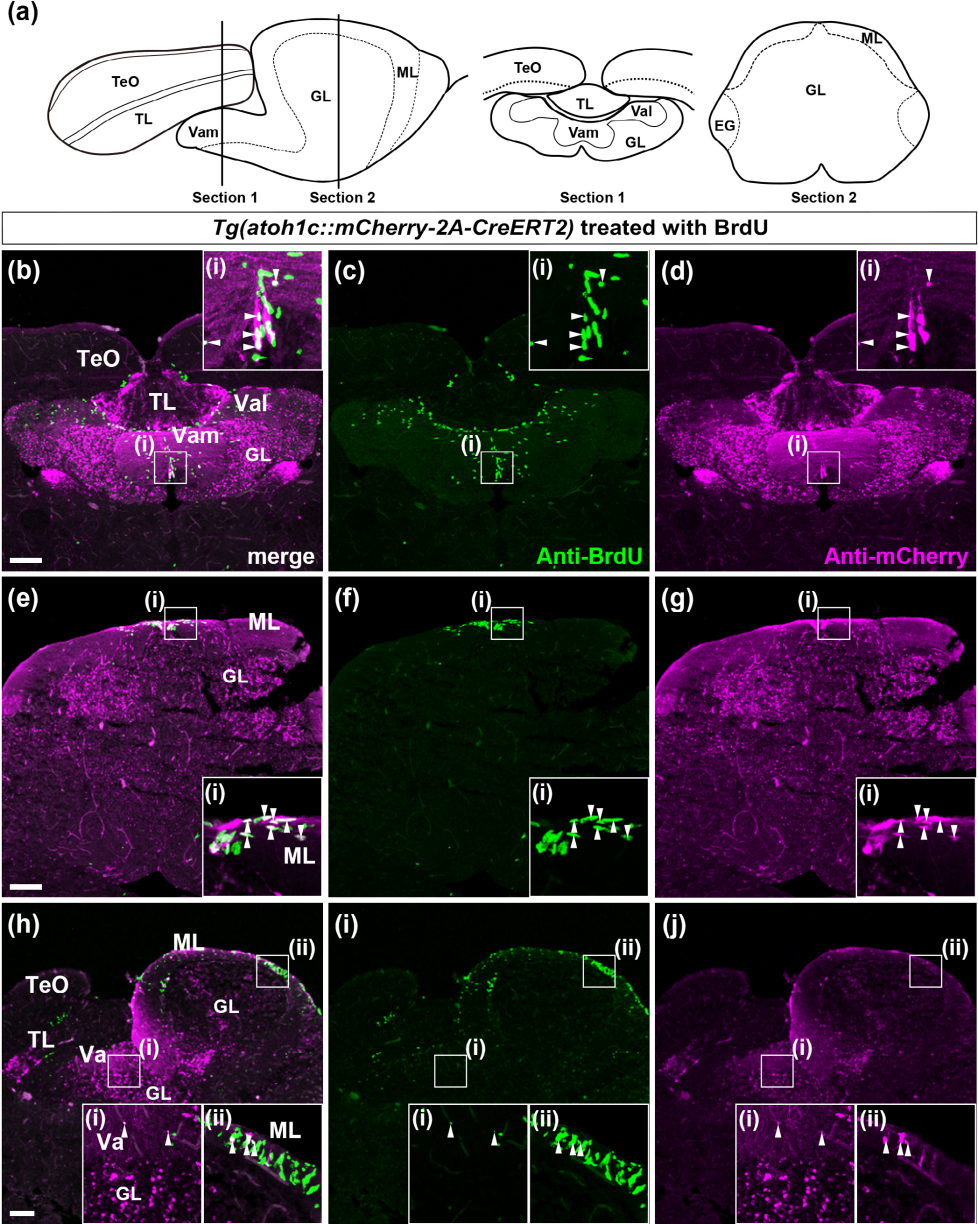
Comparison between proliferating cells and *atoh1c*‐expressing neural progenitors. Adult *Tg*(*atoh1c::mCherry‐2A‐CreERT2*) fish were treated with BrdU for 1 h and immediately killed (*n* = 4). (a) Images of analyzed sections (identical to Figure 3a). Cross sections (b–d: section 1, TL/Va level, e–g: section 2, CCe level) and a sagittal section (h–j) were analyzed by immunostaining with anti‐BrdU and mCherry. Higher magnification views of the boxed areas in the insets. Arrowheads indicate BrdU^+^ mCherry^+^ cells. Scale bars: 100 μm in b (applies to b–d), 100 μm in e (applies to e–g), 100 μm in h (applies to h–j).

It is known that neuroepithelial‐like progenitors, which are proliferative and express *nestin*, give rise to GCs in the adult cerebellum (Kaslin et al., 2009; Kaslin et al., 2013), and that *atoh1c* is expressed in proliferating cells (Kani et al., 2010). We compared mCherry^+^ cells with proliferating cells marked by short‐term BrdU incorporation (1‐h) (Figure 4b–d for section 1; Figure 4e–g for section 2; and Figure 4h–j for the sagittal section). As reported previously (Kani et al., 2010; Kaslin et al., 2017), BrdU^+^ cells are detected in the medial region of the ML of Va (Vam) and the CCe, with a few BrdU^+^ cells also observed in the dorsal region of the GL, close to the ML (Figure 4c, f, i). Some mCherry^+^ cells in the ML and dorsal GL are positive for BrdU incorporation (Figure 4b, e, h). This finding supports the notion that mCherry expression marks cells in transition between neural progenitors and immature GCs, where BrdU^+^ mCherry^+^ neural progenitor cells differentiate into BrdU^−^ mCherry^+^ immature GCs.

### 3.3 GCs derived from *atoh1c*‐expressing cells contribute to GC regeneration in the adult cerebellum

**FIGURE 5:**
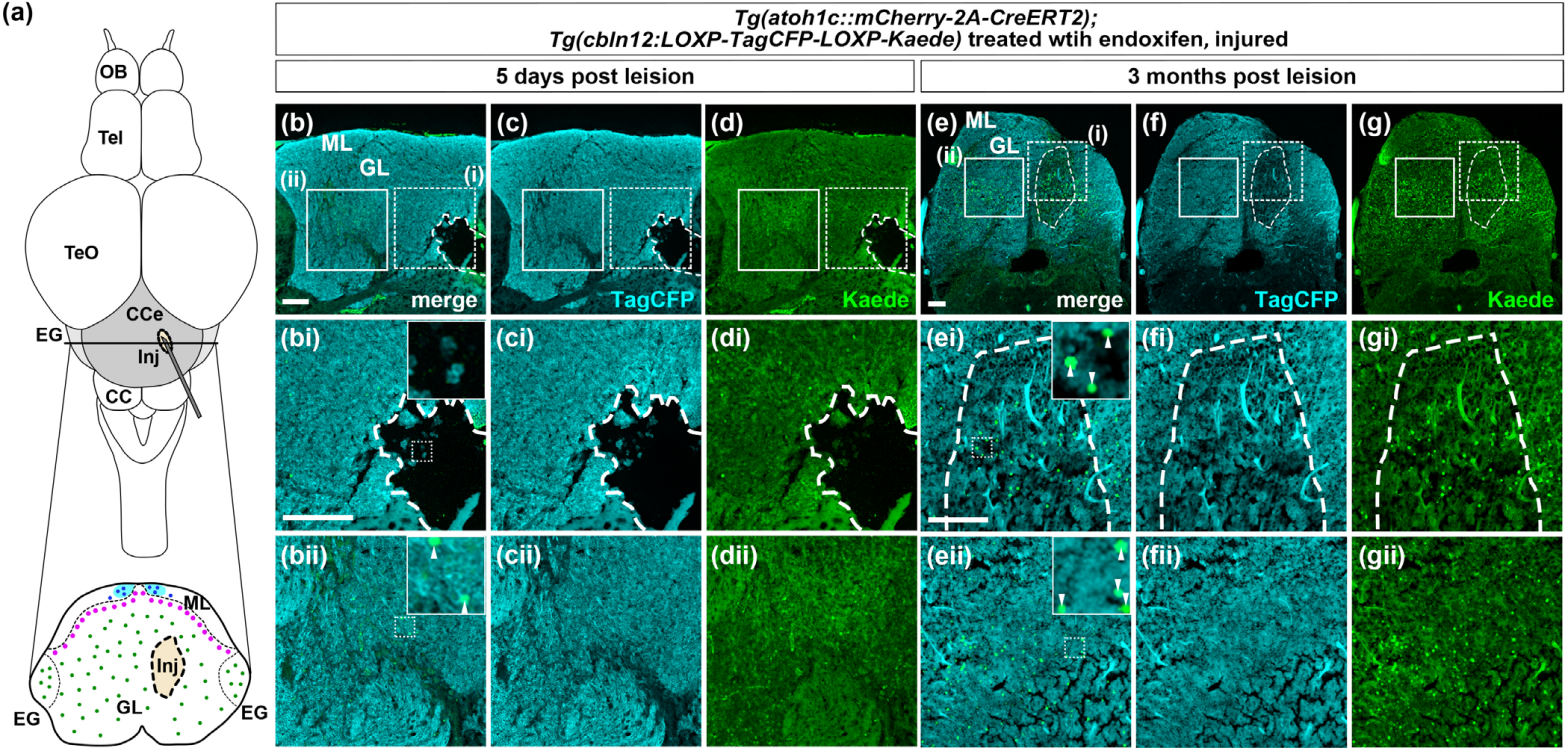
Granule cells are generated from *atoh1c*‐expressing neural progenitors during regeneration in adult zebrafish. (a) Schematic overview of the injury model. A needle was inserted from posterior to anterior in the cerebellum of the adult fish to create an injury (Inj). (b–d, e–g) Expression of TagCFP and Kaede in adult *Tg*(*atoh1c::mCherry‐2A‐CreERT2*);*Tg*(*cbln12:LOXP‐TagCFP‐LOXP‐Kaede*) fish at 5 days (b–d) or 3 months (e–g) post lesion (*n =* 3 each condition). Cross sections at the CCe level (section 2 in Figure 3a). Injured areas are outlined by dotted lines. (bi–gi, bii–gii) Higher magnification views of the dotted boxed areas (injured region) and solid boxed areas (control region) in b–g. Insets show higher magnification view of the boxed areas of in bi, bii, ei, and eii. Arrowheads indicate Kaede^+^ GCs. Scale bars: 100 μm in b (applies to b–g), 100 μm in e (applies to e–g), 100 μm in bi (applies to bi–di, bii–dii), 100 μm in ei (applies to ei–gi, eii–gii).

After mechanical injury, GCs have been shown to recover in the adult cerebellum (Kaslin et al., 2017). We next examined whether GCs differentiated from *atoh1c*‐expressing cells contribute to GC regeneration. Sixteen hours after endoxifen treatment of the lineage‐tracing Tg adult fish, a needle was inserted to create an injury in the GL of the CCe (Figure 5a). Five days post‐lesion, Kaede^+^ GCs were present in the non‐injured area of the GL but absent in the injured area (Figure 5d, di, dii). In contrast, in 3 months post‐lesion cerebella, Kaede^+^ GCs were observed in both non‐injured (Figure 5eii, fii, gii) and injured areas of the GL (Figure 5ei, fi, gi). These data suggest that GCs differentiated from *atoh1c*‐expressing neural progenitors in the adult cerebellum migrate to the lesion site and regenerate the GL.

### 3.4 TGF‐β signaling is involved in the generation of *atoh1*‐expressing neural progenitor cells

**FIGURE 6:**
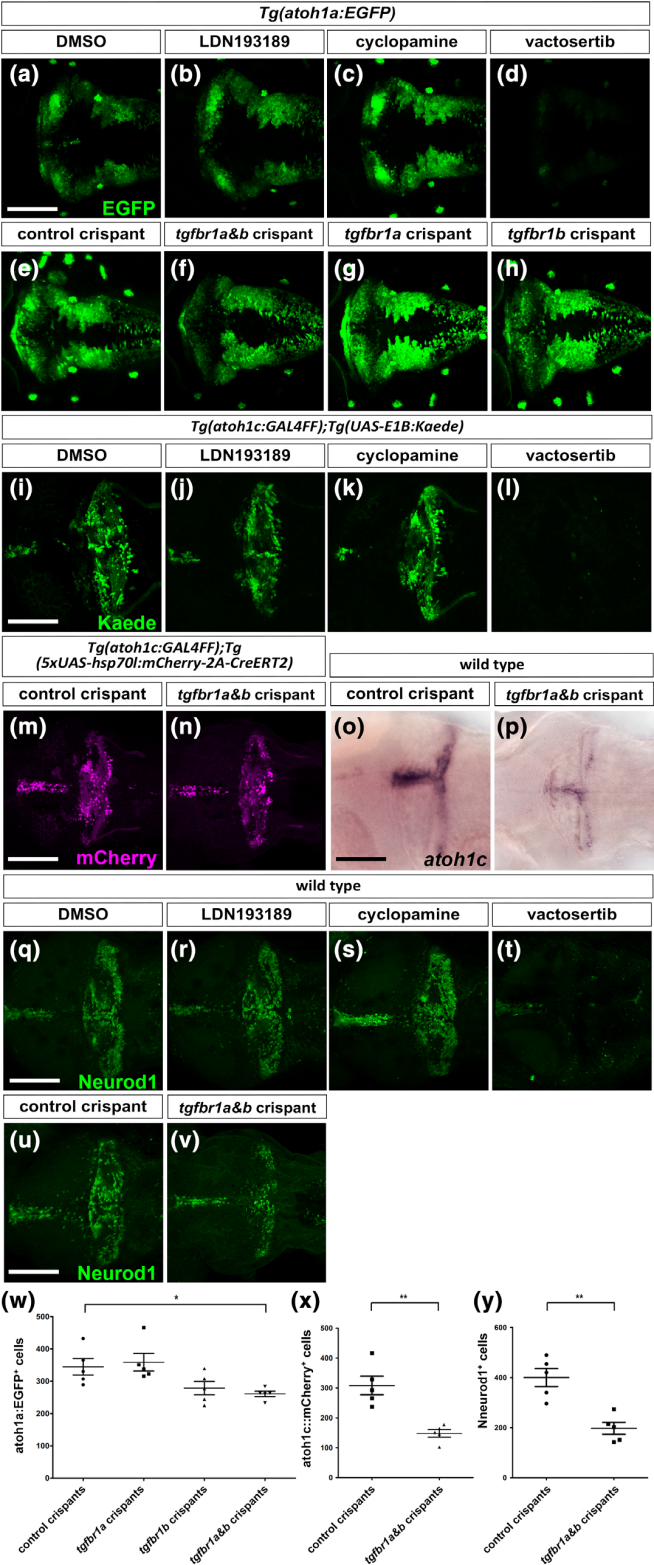
Transforming growth factor‐β (TGF‐β) signaling is required for generation of *atoh1*‐expressing neural progenitors and granule cells in larval cerebellum. (a–d) Expression of EGFP in 5‐dpf *Tg*(*atoh1a:EGFP*) larvae treated with DMSO, BMP signal inhibitor DN193189, Shh signal inhibitor cyclopamine, or TGF‐β inhibitor vactosertib after 2 dpf (*n* = 5 for each condition). (e–h) Expression of EGFP in 5‐dpf Tg larvae that received Cas9 mRNA, tracrRNA, and crRNA targeting *tgfbr1a*, and/or *tgfbr1b* (crispants) (*n* = 5 for each condition). crRNA for zebrafish *mitfb* gene was used as a control. (i–l) Expression of Kaede in 5‐dpf *Tg*(*atoh1c:GAL4FF*);*Tg*(*UAS‐E1B:Kaede*) larvae treated with DMSO, DN193189, cyclopamine, or vactosertib after 2 dpf (*n* = 5 for each condition). (m, n) Expression of mCherry in 5‐dpf Tg control and *tgfbr1a;tgfbr1b Tg*(*atoh1c::mCherry‐2A‐CreERT2*) crispants (*n* = 5 for each condition). (o, p) Expression of *atoh1c* in control and *tgfbr1a;tgfbr1b* crispants (*n* = 7 for each condition). (q–t) Expression of Neurod1 in 5‐dpf larvae treated with DMSO, DN193189, cyclopamine, or vactosertib after 2 dpf (*n* = 5 for each condition). (u, v) Expression of Neurod1 in control and *tgfbr1a;tgfbr1b* crispants (*n* = 5 for each condition). (w–y) Number of atoh1a:EGFP^+^ cells (w), atoh1c::mCherry(−2A‐CreERT2)^+^ cells (x), and Neurod1^+^ cells (y) in the cerebellum of larvae that received CRISPR/Cas9. One‐way analysis of variance with Tukey's post hoc test. *p < .05, **p < .01. Scale bar: 100 μm in a (applies to a–h), 100 μm in i (applies to i–l), 100 μm in m (applies to m, n), 100 μm in o (applies to o, p), 100 μm in q (applies to q–v).

To investigate the signaling pathways involved in the generation and/or maintenance of *atoh1*‐expressing cells, we used pathway‐specific inhibitors. These included LDN193189 (an inhibitor of BMP signaling) (Cannon et al., 2010), cyclopamine (an inhibitor of Shh signaling) (Neumann et al., 1999), and vactosertib (an inhibitor of TGF‐β signaling) (Jin et al., 2014). We validated each inhibitor's specificity by treating embryos and assessing the phenotypic effects (Figure S1). LDN193189 treatment induced football‐shaped phenotypes at the tailbud stage, indicating dorsalization due to BMP signal inhibition. Cyclopamine treatment resulted in U‐shaped somites, a characteristic feature of muscle pioneer deficiency, reflecting the inhibition of Shh signaling (Lewis et al., 1999). Vactosertib treatment expanded the dorsal blastoderm margin at the bud stage and induced cyclopia, with loss of mesoderm and endoderm during the pharyngula period, consistent with inhibition of Nodal (a TGF‐β ligand) signaling (Gritsman et al., 1999). Next, we applied these inhibitors to 2‐dpf larvae and examined their effects on *atoh1* expression and GC generation. The *Tg*(*atoh1a:EGFP*) line was used to monitor *atoh1a* expression (Kani et al., 2010), and *Tg*(*atoh1c:GAL4FF*);*Tg*(*UAS‐E1B:Kaede*) (referred to as *Tg*(*atoh1c::Kaede*)) lines were used for *atoh1c* expression (Kidwell et al., 2018). Only vactosertib abolished atoh1a:EGFP expression (Figure 6a–d), as well as atoh1c::Kaede expression and the GC marker Neurod1 (Figure 6i–l, q–t). Vactosertib is reported to specifically inhibit Acvr1b (Alk4) and Tgfbr1 (Alk5) (Jin et al., 2014), which act as receptors for TGF‐β/Nodal‐family ligands (Weiss & Attisano, 2013). Zebrafish have two *tgfbr1* genes: *tgfbr1a* (*alk5a*) and *tgfbr1b* (*alk5b*) (ZFIN, https://zfin.org). We generated *tgfbr1a/tgfbr1b* crispants via Cas9 protein, tracrRNA, and *tgfbr1a/tgfbr1b* crRNA injection. Among these crispants, *tgfbr1a* was more strongly mutated, but *tgfbr1b* exhibited weaker mutations (Figure S2). Despite this, they displayed pericardial edema, similar to *tgfbr1a*;*tgfbr1b* mutants, albeit milder (Boezio et al., 2020). Single *tgfbr1a* or *tgfbr1b* crispants did not reduce atoh1a:EGFP expression, but double *tgfbr1a*;*tgfbr1b* crispants showed a significant reduction in atoh1a:EGFP^+^ cells in the cerebellum at 5 dpf (Figure 6e–h, w). Additionally, *tgfbr1a*;*tgfbr1b* crispants showed reduced expression of *atoh1c*, atoh1c::mCherry‐2A‐CreERT2, and Neurod1^+^ GC cells at 5 dpf (Figure 6m–p, u, v, x, y). These results indicate that TGF‐β signaling is essential for the generation of *atoh1a/c*‐expressing neural progenitors and subsequent GC production in early‐stage larvae.

**FIGURE 7:**
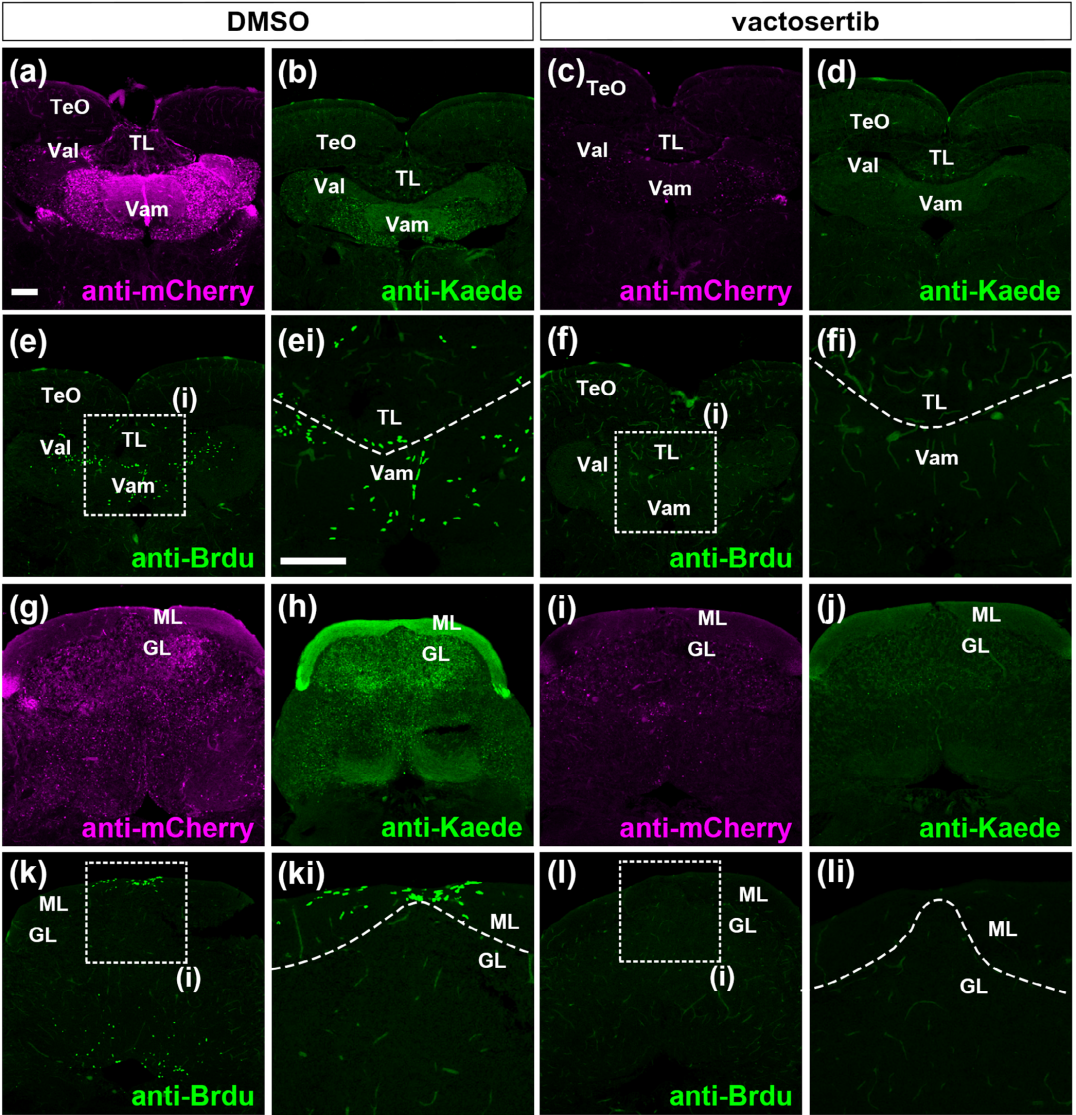
Transforming growth factor‐β (TGF‐β) signaling is required for maintenance of proliferating cells, *atoh1* expression, and generation of granule cells in the adult cerebellum. Adult *Tg*(*atoh1c::mCherry‐2A‐CreERT2*);*Tg*(*cbln12:LOXP‐TagCFP‐LOXP‐Kaede*) fish were treated with DMSO or vactosertib, followed by endoxifen 24 h later (*n* = 4). They were then maintained in system water containing either DMSO or vactosertib for 5 days, after which BrdU treatment was administered for 1 h. mCherry (a, c, g, i) and Kaede (b, d, h, j) expression, along with BrdU incorporation (e, ei, f, fi, k, ki, l, li), were analyzed by immunostaining. Cross sections (a–f, section 1 TL/Va level; g–l section 2 CCe level). (ei, fi, ki, li) Higher magnification views of the dotted boxed areas of e, f, k, l. Dotted lines indicate the boundary between TL and Va in ei and fi; between ML and GL in ki and li. Scale bars: 100 μm in a (applies to a–e, f, g–k, and l), 100 μm in ei (applies to ei, fi, ki, li).

We further investigated the role of TGF‐β signaling in adult GC neurogenesis. Lineage‐tracing Tg adult fish were treated with vactosertib or DMSO (control) for 6 days, with endoxifen administered on the second day. We analyzed mCherry^+^ neural progenitors/immature GCs and Kaede^+^ differentiated GCs (Figure 7a–d for section 1 and Figure 7g–j for section 2). While vactosertib treatment did not alter cerebellar morphology, it disrupted mCherry and Kaede expression in the Va and CCe (Figure 7c, d for the Va; Figure 7i, j for the CCe). We also assessed BrdU incorporation by treating the fish with BrdU during vactosertib exposure (Figure 7e, ei, f, fi for section 1; Figure 7k, ki, l, li for section 2). Vactosertib treatment impaired BrdU incorporation in the Va and the ML of the CCe (Figure 7f, fi for section 1; Figure 7l, li for section 2). These findings indicate that TGF‐β signaling is essential for the generation or maintenance of *atoh1c*‐expressing cells, proliferating neural progenitors, and subsequent GC differentiation in the adult cerebellum.

## 4 DISCUSSION

The *atoh1*‐family genes are expressed in the cerebellum of zebrafish from the larval stage through adulthood (Adolf et al., 2004; Chaplin et al., 2010; Kani et al., 2010). GCs are continuously produced throughout the zebrafish's lifespan (Kani et al., 2010; Kaslin et al., 2009; Kaslin et al., 2013; Zupanc et al., 2005). This study demonstrates that at least some GCs are generated from *atoh1c*‐expressing cells throughout the lifespan of zebrafish (Figures 2, 3). This finding suggests that GC production in adults (adult neurogenesis) is governed by mechanisms similar to those that control initial GC production, extending the processes established during early GC generation.

### 4.1 *atoh1c* is transiently expressed in neural progenitor cells initiating GC differentiation

In this study, we used a GAL4‐UAS system to express CreERT2 for lineage tracing. *CreERT2* mRNA was also expressed in the CRL, but mCherry, translated from the same mRNA as CreERT2, exhibited weak expression in the CRL and was more prominently expressed in more differentiated cells in larvae (Figure 1). Similarly, mCherry was detected in cells located in the GL rather than in the ML of the adult cerebellum, where *atoh1c* is expressed (Figures 1, 3). As the Gal4‐UAS system introduces an additional step where GAL4FF is first expressed and subsequently binds to the UAS to drive mCherry and CreERT2 expression, this results in a delay compared with the direct expression of mCherry and CreERT2 from the enhancer. Consequently, mCherry and CreERT2 expression occurs later than that of *atoh1c*, and recombination takes place afterwards. If *atoh1c* were continuously expressed in proliferating neural progenitor cells in the CRL, mCherry would also be strongly expressed there; however, this is not observed. These data suggest that *atoh1c* is expressed in neural progenitor cells at the onset of GC differentiation. Mutant analyses further show that *atoh1c* is essential for the differentiation of most GCs but not the generation of neural progenitor cells (Kidwell et al., 2018), indicating that *atoh1c*‐expressing cells among neural progenitors are likely committed to differentiation.

### 4.2 Developmental process of GC neurogenesis (Figure 8)

**FIGURE 8:**
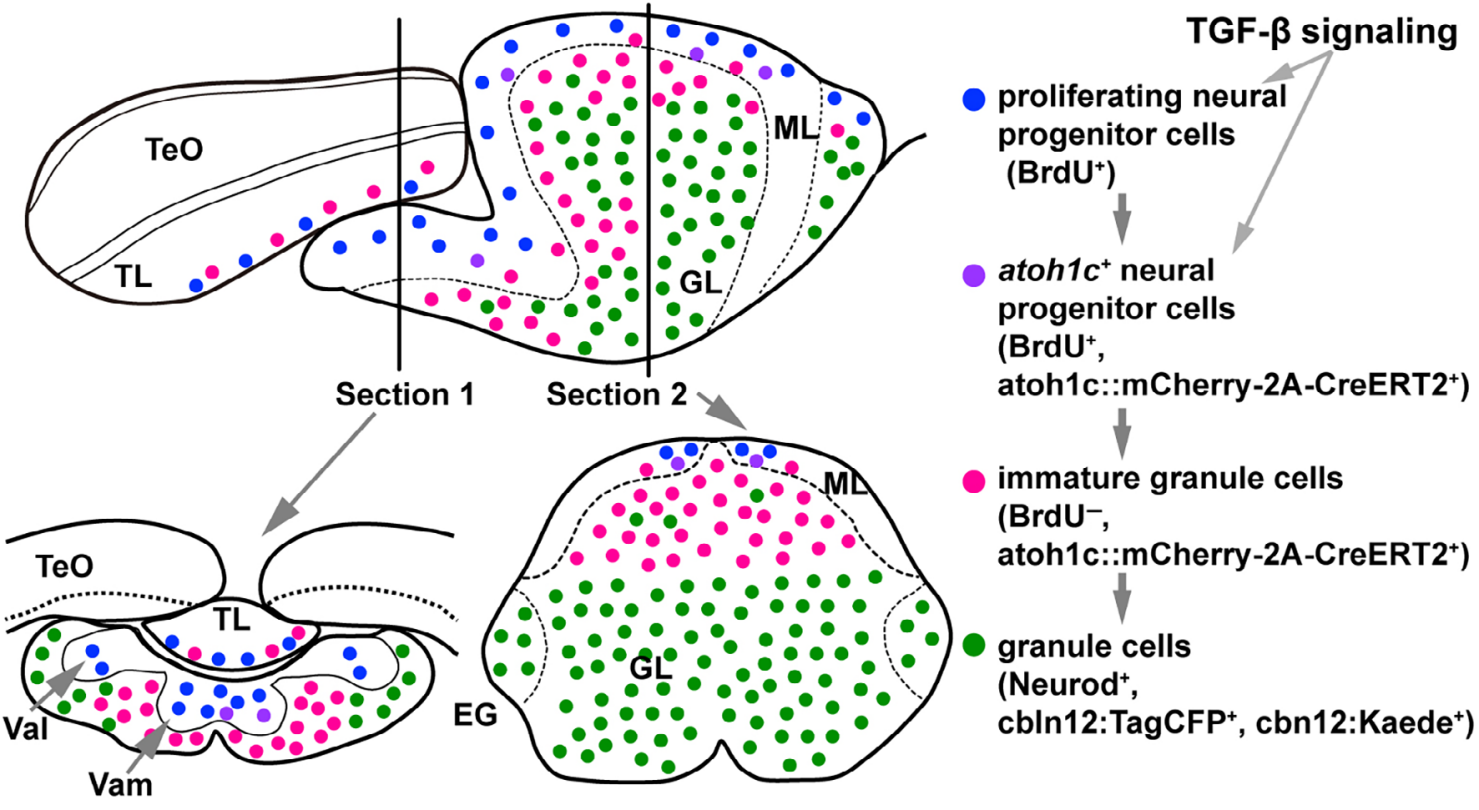
Schematic of a model for granule cell development in adult fish. Proliferating neural progenitor cells that generate GCs are located in the ML (indicated by blue circles). Among these, some cells expressing *atoh1c* start to differentiate (indicated by purple circles) and migrate from the ML (indicated by pinkish circles) to the GL, where they localize as mature GCs (indicated by green circles). A similar phenomenon is observed in the Va. In the TL, GCs are produced from *atoh1c*‐expressing cells in larvae, but production is limited in adults. Details are provided in Section 4, Discussion.

It has been shown that some *atoh1a*‐, *atoh1b*‐, or *atoh1c*‐expressing cells in the larval and adult cerebellum can incorporate BrdU, indicating that a subset of *atoh1*‐expressing progenitors are proliferative (Kani et al., 2010). Consistent with this, some *atoh1c::mCherry‐2A‐CreERT2* ^+^ cells also incorporated BrdU in the adult cerebellum (Figure 4). However, BrdU^+^ but *atoh1c* reporter‐negative cells were also observed in the medial ML of the adult cerebellum, which may include *nestin*‐expressing neuroepithelial cells (Kaslin et al., 2009; Kaslin et al., 2013; Kaslin et al., 2017). These findings suggest that *atoh1c* ^+^ cells likely originate from proliferative neural progenitors, potentially including *nestin*‐expressing neuroepithelial cells. Similarly, in mice, two distinct regions are reported in the CRL: one exhibits high levels of the proliferating marker Mki67 and low *Atoh1* expression, whereas the other shows low Mki67 and high *Atoh1* expression (Butts et al., 2024). In both teleosts and mammals, actively proliferating neural progenitor cells are present in the CRL of larvae and the medial ML of the adult cerebellum. Upon *atoh1* expression, these progenitors stop proliferating and begin differentiation. In zebrafish, this process continues throughout life, with GCs differentiated from *atoh1*‐expressing cells migrating throughout the GL within 5 days. Our findings also indicate that in the TL, GCs are generated from *atoh1*‐expressing cells during the larval stage, but only very weakly in adulthood.

### 4.3 Regeneration of GCs

It has been reported that, in response to injury, *nestin*‐expressing neuroepithelial cells are activated, proliferate more, and produce GCs to repair lesions (Kaslin et al., 2017). This study further shows that GCs derived from *atoh1c*‐expressing cells also contribute to regeneration, restoring GCs in injured areas (Figure 5). Hence, as in adult neurogenesis, proliferating progenitor cells expressing *atoh1c* differentiate and migrate to injured areas. We did not observe a significant increase in *atoh1c* ^+^ lineage cells near injuries, possibly due to differences in injury methods or specific unexamined areas where *atoh1*‐expressing progenitors might be activated. Further research is needed to clarify how *atoh1c*‐expressing progenitor cells respond to injury and how newly generated GCs migrate to lesion sites.

To date, there have been no reports of neurogenesis or regeneration of GCs in adult mammals. Elucidating the molecular mechanisms underlying these processes in zebrafish may help in developing methods to regenerate granule cells in mammals, including humans.

### 4.4 Role of TGF‐β signaling in GC neurogenesis

Several signaling pathways have been implicated in the generation and/or maintenance of neural progenitors for GCs in zebrafish, including BMP, Shh, and Fgf pathways. Inhibition of Shh signaling with cyclopamine did not repress *atoh1a* or *atoh1c* reporter expression or Neurod1^+^ GC generation at the larval stage (Figure 6), suggesting a minimal role for Shh in early GC development. This is consistent with the absence of *shha/b* expression in PCs and of the Shh target gene *ptch1* in the larval cerebellum (Chaplin et al., 2010; Takeuchi et al., 2017). Genetic ablation of *ptch1*, which normally inhibits Shh signaling, leads to medulloblastoma‐like tumors in the adult zebrafish brain, though the cerebellum is not significantly affected (Casey et al., 2024). Additionally, *shha* promoter activity is absent in the adult cerebellum (Wullimann & Umeasalugo, 2020), suggesting that Shh signaling likely has a limited role in cerebellar development in both larvae and adults. BMP ligands are expressed near neural progenitors in the CRL of larvae (Chaplin et al., 2010). However, inhibition of BMP signaling with LDN193189 did not affect *atoh1a/c* reporter expression or Neurod1^+^ GC generation in larvae (Figure 6). BMP signaling is reported to be involved in stability of Atoh1 protein (Zhao et al., 2008) and migration of GCs (Rook et al., 2024). Therefore, BMP signaling may play a role in terminal differentiation and migration of GCs but not in production of GCs.

In contrast, inhibition of TGF‐β signaling through vactosertib led to a strong reduction in *atoh1a/c* reporter expression and Neurod1^+^ GC generation. CRISPR/Cas9‐mediated knockout of *tgfbr1a* and *tgfbr1b* genes partially recapitulated the vactosertib‐treated phenotypes (Figure 6). Additionally, vactosertib treatment suppressed *atoh1c* reporter activity and GC neurogenesis from *atoh1c* ^+^ progenitors (Figure 7), highlighting a previously unrecognized role of TGF‐β in *atoh1* ^+^ progenitor generation. Inhibition of TGF‐β signaling also reduced BrdU incorporation in proliferating neural progenitors in the adult cerebellum (Figure 7). It remains unknown whether TGF‐β signaling specifically controls the generation/maintenance of *atoh1* ^−^ (BrdU^+^) or *atoh1* ^+^ neural progenitors, or both (Figure 8). Targeted inhibition in specific cell populations (e.g. *nestin* ^+^ or *atoh1* ^+^ cells) would help to clarify this. It is also unclear which TGF‐β ligands function in this process and whether they act locally in the cerebellum or remotely via cerebrospinal fluid. Future studies identifying TGF‐β ligands and downstream genes would address these questions.

Whether the TGF‐β signaling‐dependent GC production mechanism identified here is specific to teleosts or a conserved feature among vertebrates remains an open question for future studies. However, this research sheds light on the mechanisms underlying the production of GCs—one of the most abundant neuron types in the brain—providing valuable insights into neuronal development.

## AUTHOR CONTRIBUTIONS

J. C. W., T. S., and M. H. designed the research; J. C. W. and M. H. performed the research, analyzed the data, and wrote the paper.

## FUNDING INFORMATION

Japan Society for the Promotion of Science, Grant/Award Number: 18H02448 and 23K2389.

## CONFLICT OF INTEREST STATEMENT

The authors declare no competing or financial interests.

## Supporting information


**FIGURE S1.** Effects of chemical inhibitors on zebrafish development. Images of AB embryos at 10 hpf (a–c) and larvae at 1 dpf (d–g) treated with DMSO (a, *n* = 15/15; d, *n* = 15/15), the Bmp signaling inhibitor LDN193189 (b, *n* = 10/10; e, *n* = 6/6, 4 dead), the Shh signaling inhibitor cyclopamine (f, *n* = 10/10), or the transforming growth factor‐β signaling inhibitor vactosertib (c, *n* = 29/29; g, *n* = 27/27, 2 dead). Scale bars: 100 μm in a (applies to a–c), 100 μm in d (applies to d–g).

**FIGURE S2.** Effects of CRISPR/Cas9. Genotyping of crispants. Target genomic regions of the *tgfbr1a* and *tgfbr1b* genes were amplified from 5‐dpf larvae, including two control wild‐type samples and five *tgfbr1a;tgfbr1b* crispants, and analyzed by acrylamide gel electrophoresis. w, wild‐type; n, negative control (only buffer); m, 100‐bp ladder marker.

## Data Availability

The data that support the findings of this study are available from the corresponding author upon reasonable request.
