# Supplementary figures and images for: Transforming growth factor‐β‐mediated regulation of atoh1‐expressing neural progenitors is involved in the generation of cerebellar granule cells in larval and adult zebrafish
Source: Dev Growth Differ. 2025 Feb 27;67(3):149–64. doi: 10.1111/dgd.70002 (PMC11997739; doi:10.1111/dgd.70002)

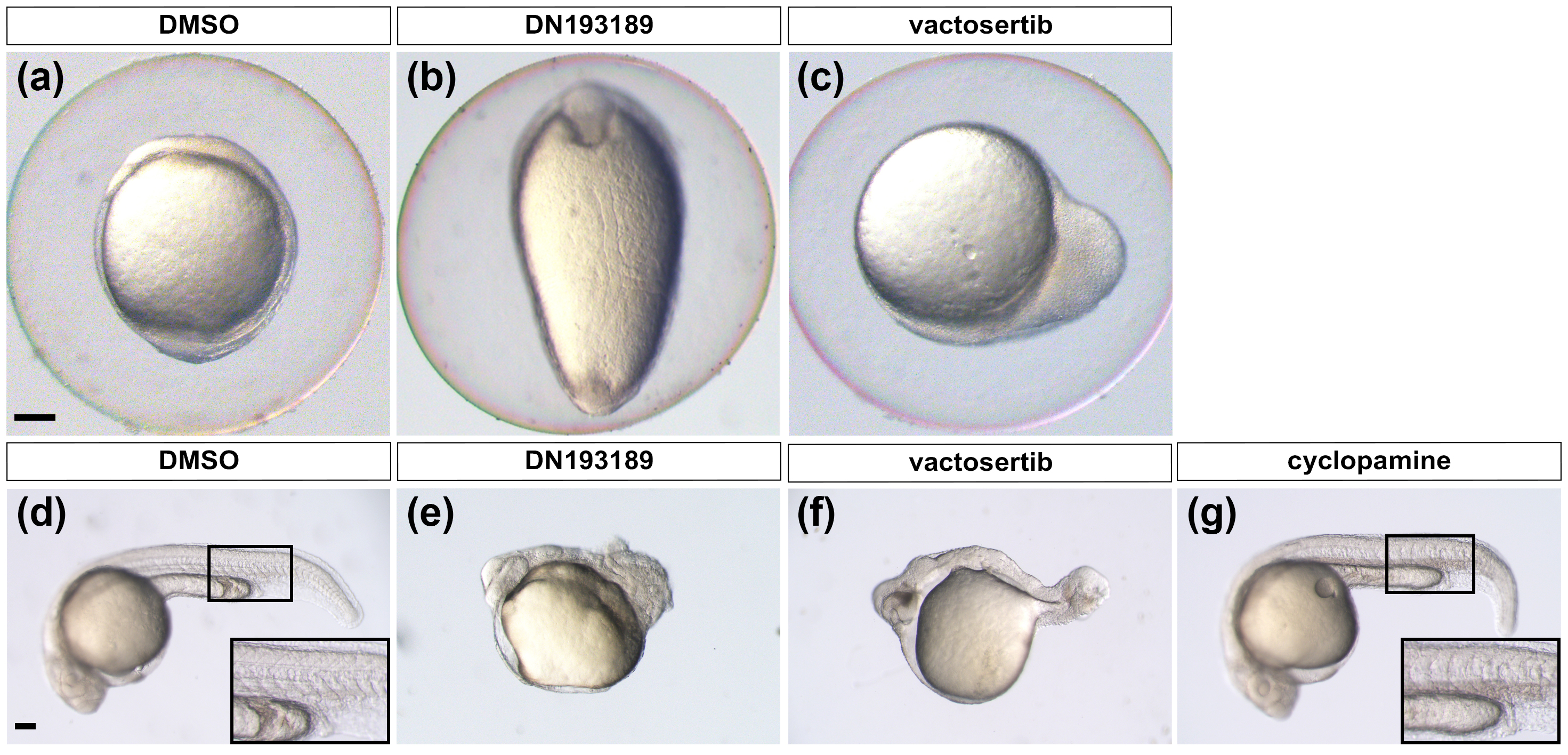

Supplement: Supplementary file 1 — FIGURE S1. Effects of chemical inhibitors on zebrafish development. Images of AB embryos at 10 hpf (a–c) and larvae at 1 dpf (d–g) treated with DMSO (a, n = 15/15; d, n = 15/15), the Bmp signaling inhibitor LDN193189 (b, n = 10/10; e, n = 6/6, 4 dead), the Shh signaling inhibitor cyclopamine (f, n = 10/10), or the transforming growth factor‐β signaling inhibitor vactosertib (c, n = 29/29; g, n = 27/27, 2 dead). Scale bars: 100 μm in a (applies to a–c), 100 μm in d (applies to d–g). [file DGD-67-149-s002.jpg]

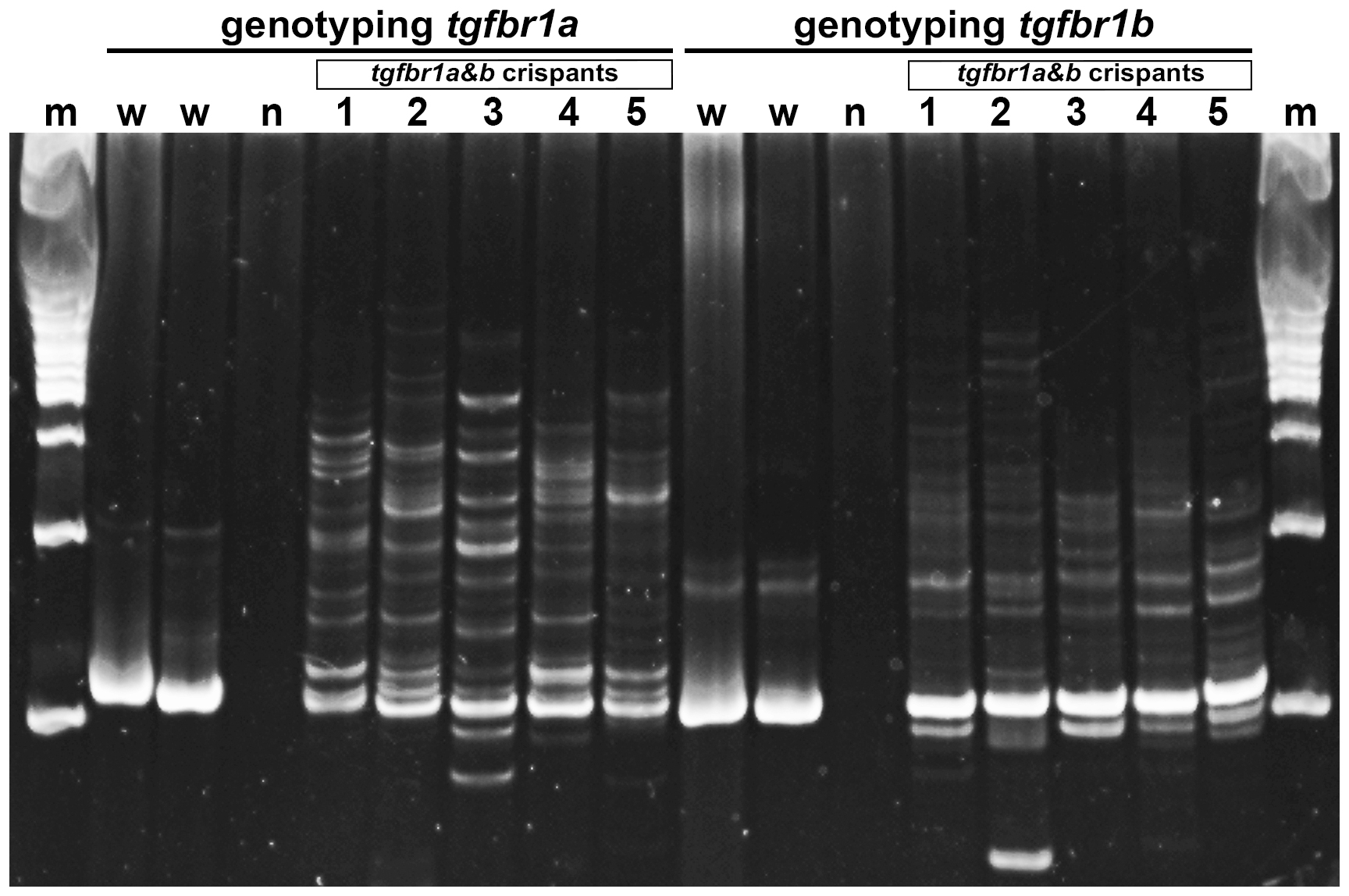

Supplement: Supplementary file 2 — FIGURE S2. Effects of CRISPR/Cas9. Genotyping of crispants. Target genomic regions of the tgfbr1a and tgfbr1b genes were amplified from 5‐dpf larvae, including two control wild‐type samples and five tgfbr1a;tgfbr1b crispants, and analyzed by acrylamide gel electrophoresis. w, wild‐type; n, negative control (only buffer); m, 100‐bp ladder marker. [file DGD-67-149-s001.jpg]
